# OSERR: an open-source standalone electrophysiology recording system for rodents

**DOI:** 10.1038/s41598-020-73797-4

**Published:** 2020-10-12

**Authors:** Ning Cheng, Kartikeya Murari

**Affiliations:** 1 Alberta Children’s Hospital Research Institute (ACHRI), Cumming School of Medicine, University of Calgary, 3300 Hospital Dr., N.W., Calgary, AB T2N 4N1 Canada; 2 Department of Electrical and Computer Engineering, Schulich School of Engineering, University of Calgary, 2500 University Dr., N.W., Calgary, AB T2N 1N4 Canada

**Keywords:** Neuroscience, Neurophysiology, Electrophysiology

## Abstract

Behavioral assessment of rodents is critical for investigation of brain function in health and disease. In vivo neurophysiological recordings are powerful tools to mechanistically dissect neural pathways that underlie behavioral changes, and serve as markers for dynamics, efficacy and safety of potential therapeutic approaches. However, most in vivo recording systems require tethers or telemetry receivers, limiting their compatibility with some behavioral tests. Here, we developed an open-source standalone electrophysiology recording system for rodents (OSERR). It is a tether-free, standalone recording device with two channels, a reference and a ground, that acquires, amplifies, filters and stores data all in itself. Thus, it does not require any cable or receiver. It is also compact and light-weight, and compatible with juvenile mice, as well as multiple recording modalities and standard electrode implantation methods. In addition, we provide the complete design of hardware, and software for operation. As an example, we demonstrated that this standalone system, when configured with a bandwidth of 1–120 Hz and gain of 1000, successfully collected EEG signals during induced seizure, extended recording, anesthesia, and social interactions in mice. The design of this system is practical, economical, and freely available. Thus, this system could enable recording of brain activity during diverse behavioral assays in a variety of arenas and settings, and allow simultaneous recordings from multiple subjects to examine social behaviors. Importantly, with the open-source documentation, researchers could customize the design of the system to their specific needs.

## Introduction

Methods for in vivo electrophysiological recording in rodents, including EEG, local field potential, single or multiple unit recording, and intracellular recording, have been routinely used in neuroscience research and provided invaluable information on diverse processes in the brain, from single neuron firing to long-range functional connectivity and network synchrony^1–6^. Clinically, they have been increasingly utilized as biomarkers for neurological conditions^7–9^. On the other hand, behavioral tests using rodent models have become increasingly sophisticated and indispensable for studying brain function in both normal and diseased states^10–12^. Importantly, behavioral readout has been a key component in testing the efficacy of potential therapeutic approaches^13–16^.

Combining in vivo electrophysiological recording with behavioral tests provides information on brain functions related to the ongoing behavior, and thus has opened a range of possible investigations on the cellular and populational activities and the dynamic nature of the neural substrate underlying behaviors^17–22^. However, most electrophysiological recording systems are tethered. While offering important advantages such as high channel count up to many thousands^23,24^, tethered systems limit the movement of the subjects, and may affect behavioral phenotype. In addition, other potential problems are also associated with tethered systems, including susceptibility to electromagnetic interference and movement artifacts, as well as the risk that tethered animals may injure themselves or disrupt the recording by pulling on the cable^25^. Radio telemetry systems allow test subjects to move freely^26–32^, and both tethered and telemetry systems offer the advantage of real-time data streaming for visualization and closed-loop headstage communication. However, the requirement of receivers by telemetry systems could render recording during certain behavioral tests challenging, such as the ones requiring large and complicated arenas with obstacles that may impair signal transmission. Design of standalone recording systems called neurologgers or logging headstages, which do not need tethers or receivers, have been reported for use in humans^33^, pigeons^34^, and rodents^35,36^. However, these devices are either too large for use with rodents or are proprietary designs. Commercial devices for use with rodents are also available, but they are difficult to customize and can be costly.

Here, we developed such a system that is compact and light-weight, and compatible with multiple recording modalities. Importantly, it is a tether-free, standalone recording device that acquires, amplifies, filters and stores data all in itself, and thus does not require any cable or receiver. We named it OSERR, for open-source standalone electrophysiology recording system for rodents. It can be connected to animals using standard electrode implantation methods, and can be used in standard rodent home cages and a variety of behavioral arenas and settings, including interactions among multiple animals in close-to-natural situations. OSERR is practical and economical, and is being made available as an open-source tool. We further provided the design of a simple reader and graphical user interface to transfer data from the device to a computer. As a demonstration, we used the standalone system to successfully record EEG data during induced seizure, overnight recording, anesthesia, and male–female and male-male social interactions.

## Materials and methods

### Design and fabrication of OSERR

**Figure 1:**
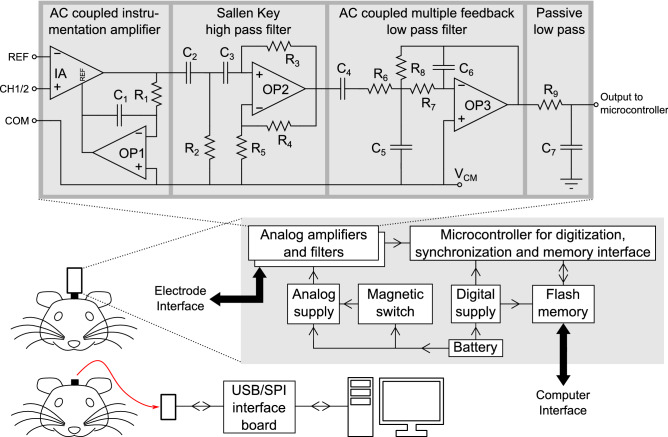
A block diagram of OSERR showing the overall architecture, details of the analog chain and the operation. The system is plugged into an implanted electrode socket on the animal for recording and data are stored in an onboard flash memory. At the end of the recording, the system is unplugged, connected to a computer through an interface board and data are read out for analysis.

Figure 1 shows a simplified block diagram of the system and details of the analog front-end. Detailed circuit schematics, printed circuit board (PCB) layouts, parts list, assembly guide, source code and operation manual can be found in the supplementary information. The design has two differential input channels with a common reference and a driven ground. The analog conditioning consists of an ac-coupled instrumentation amplifier, a Sallen-Key highpass and a multiple feedback lowpass filter, providing third order (60 dB/decade) attenuation on either side of the passband. The lowpass and highpass cutoff frequencies, and the gain can be set using specific resistor and capacitor values. Following the analog section, a microcontroller digitizes the signal to 10 bits at a firmware-programmable sampling rate and stores it in a flash memory chip. The system is powered by a 30 mAh CR927 lithium cell which is regulated down to 2.5 V and 1.2 V as the supply voltage and virtual ground level, respectively. Independent linear regulators are used for analog (amplifiers) and digital (microcontroller and memory) components. The design is implemented on three 0.6-mm-thick PCBs—one for analog components, one for digital and one for the battery—that are stacked vertically. A 6-pin male header is used to plug in the system to a mouse with implanted electrodes connected to a mating receptacle cemented to the skull.

**Figure 2:**
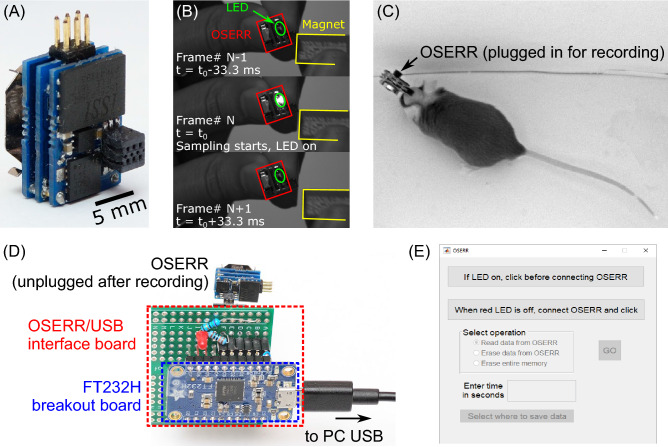
Configuration of OSERR. (**A**) A photograph of the system. It measures 13 × 8 × 8 mm^3^ and weighs 1.8 g including the battery. (**B**) Sequence of 3 consecutive frames from a video showing the synchronization procedure. A magnet is used to start acquisition which is marked by an LED flash. (**C**) A photograph of OSERR plugged into an implanted electrode socket on a mouse. (**D**) After recording, OSERR is unplugged from the animal, plugged into the OSERR/USB interface board and data are read using a MATLAB GUI (**E**).

A Hall effect switch is used to wirelessly start acquisition and storage by bringing a magnet close to the system after it is plugged into the animal. The system has a light emitting diode (LED) to synchronize recording to external stimuli or to behavioral video recording. Specifically, the system flashes an on-board LED at the start of data acquisition. This can be captured on a single frame on a camera, synchronizing the EEG acquisition with the video recording as shown in Fig. 2B. Any other recording or stimulating device with TTL pulse capabilities can be synchronized to the EEG recording via the video. For example, the pulse could drive an LED that can be captured on the video recording. Nominal analog current draw is under 150 μA while the digital current draw depends on sampling rate.

For reading out the data from the system at the end of a recording session, an interface board and graphical user interface (GUI) were developed. After recording, the system is unplugged from the animal and plugged into the interface board which connects to a computer through a USB port. The OSERR/PC interface board was developed using an FTDI 232H USB bridge chip on an Adafruit breakout board. A PCB was developed around the breakout board to convert from 3 V signalling on the FTDI 232H to 2.5 V signalling on OSERR and to add a status LED. Using the GUI, data for a user-specified duration (i.e. the length of the recording) is read into the computer at 10 Mbps. The GUI also enables erasing the flash memory prior to a subsequent recording session. For the system designed for this EEG study, the amplifiers were configured for a gain of 60 dB, leading to a full scale input of ± 1.25 mV and a least significant bit (LSB) of 2.4 μV. The passband was set from 1 to 120 Hz and the sampling rate was set to 240 Sps. A 256 Mbit memory was used, giving a 15.5 h operation time.

### Animals

Breeder C57/Bl6(J) (B6) animals were obtained from the Jackson Laboratory (ME) and the line was maintained at the mouse facility of the Cumming School of Medicine, University of Calgary. Mice were housed in a humidity- and temperature-controlled room with a 12-h light/dark cycle (light off at 7 p.m.) and were fed ad libitum. Adult B6 male and female animals aged 7–10 weeks were used. All procedures in this study were performed according to the recommendations by the Canadian Council for Animal Care. The protocol of this study was approved by the Health Sciences Animal Care Committee of the University of Calgary.

### EEG surgery, recording and data analysis

Following established methods^37,38^, anesthesia in mice was induced with inhalation of 5% isoflurane in 100% O_2_, and then maintained with 1–2% isoflurane. Mice were secured on a stereotaxic apparatus on top of a warm heating pad. Eye gel was applied to the eyes to prevent drying. The scalp was shaved and cleaned with iodine and 70% ethanol. After confirming the absence of toe pinch reflex, the skull was exposed by removing the skin on top and gently pushing aside muscles and connective tissue. Then, three small holes were drilled in the skull for differential frontal-parietal recordings. Electrodes, which are screws attached to a wire, were fixed into the holes at the following coordinates: reference electrode—left frontal cortex (AP: + 2.5 mm, ML: − 1.3 mm, relative to bregma); recording electrode—left parietal cortex (AP: − 2.2 mm, ML: − 2.5 mm); and ground (AP: + 2.5 mm, ML: + 1.3 mm). The electrodes were then soldered to a miniature connector, and the assembly was secured to the skull with dental cement. After the surgery, mice were treated with analgesic and maintained on the heating pad with O_2_ inhalation until they showed signs of ambulation. Mice were then individually housed and monitored daily. For EEG recording, OSERR was plugged into the mice under brief anesthesia by isoflurane inhalation.

For analysis, EEG data stored in OSERR’s memory were read into a computer using the interface board. Power spectral density (PSD) of the EEG was calculated using Welch's method^39^ implemented in MATLAB. EEG data were split into 2-s epochs. Periodograms were calculated for each epoch using a Hamming window and averaged to obtain the final PSD estimate at a 0.5 Hz spectral resolution. The PSD was integrated using the trapezoidal method to find the power in individual bands: delta (1–4 Hz), theta (4–8 Hz), alpha (8–13 Hz), beta (13–30 Hz), and gamma (30–100 Hz). The gamma band was further divided into low gamma (30–60 Hz) and high gamma (60–100 Hz). Time–frequency analysis was done using a short-time Fourier transform implemented in MATLAB. Power is displayed on a log scale with power in dBµ = 10log_10_(power in µV^2^).

### Synchronization between EEG and video recording

As mentioned above, a built-in LED on OSERR flashed once at the start of the acquisition of EEG signal. We captured this flash on the same camera which continuously recorded later behavioral tests. This way, we could synchronize the acquired EEG signal with the behavioral video offline, with resolution limited to the duration of a single frame of the camera we used here, which was 33 ms (30 frames/s).

### Seizure induction and recording

OSERR was plugged into the mouse under brief anesthesia by isoflurane inhalation and recording started. Following established methods^40^, intraperitoneal administration of a single high dose of kainate (45 mg/kg) was used to induce seizures. After the injection, the mouse was placed back to its home cage and recorded for another hour. The behaviors were video-taped at the same time.

### Anesthesia induction by isoflurane inhalation and EEG recording

OSERR was plugged into the mouse under brief anesthesia by isoflurane inhalation and recording started. Half an hour after the animal woke up, it was placed in an induction chamber and anesthetization was induced with inhalation of 5% isoflurane in 100% O_2_.

### Male–female and male–male social interaction and recording

OSERR was plugged into a subject male mouse as mentioned earlier and recording started. The subject was returned to his home cage. After 10 min, the lid of the subject’s home cage was removed and a female or male stranger mouse was introduced to the home cage of the subject mouse for 5 min, following established methods^41^. One subject mouse interacted with only one intruder. n = 6 for female intruder experiment and n = 5 for male intruder experiment.

## Results

### Design of hardware

**Figure 3:**
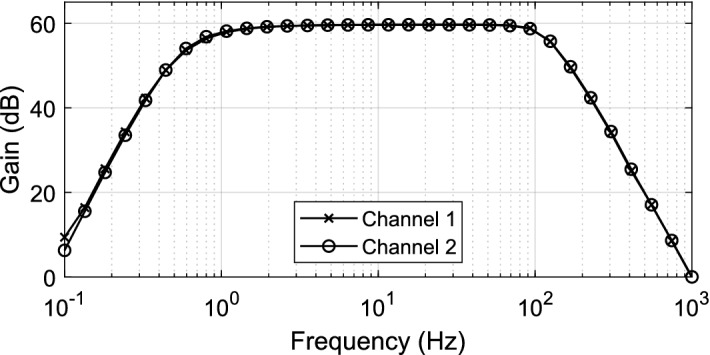
Measured transfer function for both channels of the system. Measured gain and cutoff frequencies were 59.6 dB, 0.8 Hz and 114.5 Hz, respectively.

**Figure 4:**
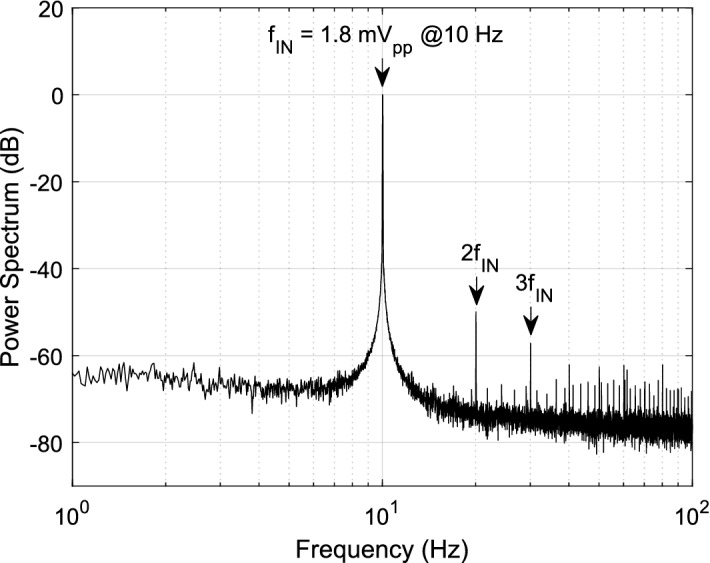
Power spectrum of the digitized signal with a 10 Hz, 1.8 mV_pp_ sinusoidal input. Total harmonic distortion was calculated to be 0.32% for the first five harmonics.

Figure 2A shows a photograph of the assembled system. It measures 13 × 8 × 8 mm^3^ and weighs 1.8 g including the battery. Figure 2B shows the synchronization procedure by a flash emitted from an on-board LED at the start of data acquisition. A sequence of 3 consecutive frames from a video illustrates that a magnet is used to start data acquisition, which is marked by an LED flash. Figure 2C shows an image of OSERR plugged into an implanted electrode socket on a mouse. Figure 2D shows OSERR connected to the USB interface board. Figure 2E shows a screenshot of the GUI used to read data into a computer. The transfer function for the analog front end was measured from 0.1 to 1000 Hz using a lock-in amplifier. The data for both channels is shown in Fig. 3. The measured gain and cutoff frequencies were 59.6 dB, 0.8 Hz and 114.5 Hz, respectively. Noise, distortion, and common mode rejection ratio (CMRR) were characterized for the overall system. Characterization data was saved in the flash memory and transferred to a computer, using the interface board and GUI, for analysis. For noise measurement, the inputs of the amplifier were shorted together. The standard deviation of the digitized data was calculated to be 0.9 LSBs which correspond to a noise of 2.3 μV referred to the input. For total harmonic distortion (THD) measurement, a 1.8 mV_pp_ 10 Hz differential sinusoid signal generated by an SRS860 lock-in amplifier (THD < 0.01%) was presented to the input. Figure 4 shows the average power spectrum of eight 1-min segments of the digitized data with the power of the fundamental normalized to 0 dB. The THD was 0.32% considering the first five harmonics. For CMRR measurement, a 180 mVpp 10 Hz was applied to both channels. The common mode gain was measured to be − 16.5 dB, giving a CMRR > 76 dB. System performance was maintained for electrode offsets of up to ± 1 V. Total current draw for the entire system was measured to be 210 μA. A comparison of OSERR and existing standalone systems are provided in Table 1.

**Table 1:** Comparison of technical specifications of some electrophysiology logging devices.

	ONEIROS^36^	MouseLog16C (Deuteron Technologies)	Neurologger 2A (Evolocus)	OSERR (this work)
Channel count	Up to 26	16	4 (2 references)	2 (1 reference)
Bandwidth	4 kHz (shared)	1 Hz–7 kHz	–	1–120 Hz
Gain	192	–	–	1000
Noise	–	2.2 µVrms	–	2.3 µVrms
Weight	5.4 g	2.88 g	2.68 g	1.8 g
Size	15 × 18 × 28 mm^3^	18 × 13 × 5 mm^3^	22 × 15 × 5 mm^3^	13 × 8 × 8 mm^3^
Battery life	Configuration dependent	100 min (32.25 kSps)	33 h (100–400 Sps)	50 h (240 Sps)
Synchronization	–	RF transciever	IR receiver	LED

‘–’ indicates the value was not specified.

To provide a few examples of potential usages that OSERR could be applied to, we recorded surface EEG using OSERR during the following different biological conditions, including social interactions.

### Detection of seizure activity in the brain by the standalone system

**Figure 5:**
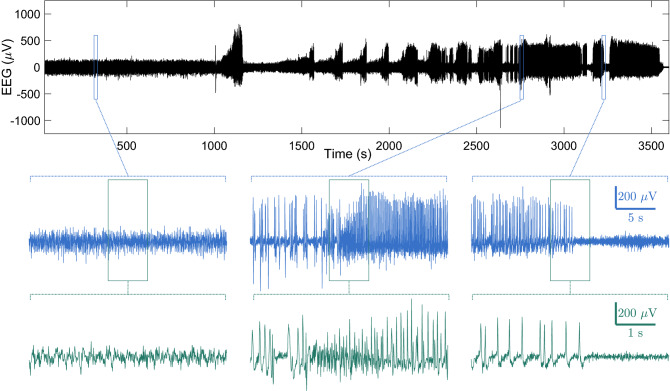
Detection of seizure activity in the brain by OSERR. At the beginning of the recording shown, a single high dose of kainate (45 mg/kg) was intraperitoneally administrated to induce seizures. Segments of distinct EEG activities during the recording are shown at three different time scales.

Brain activity during seizures is commonly recorded by EEG. To test whether the standalone system could record seizure activity in the brain, we recorded EEG using OSERR and artificially induced a seizure. After intraperitoneal administration of a single high dose of kainate (45 mg/kg) to the mouse, we observed large-amplitude discharge occurring in episodes interleaved by periods of relatively small-amplitude EEG activity (Fig. 5). Closer examination of the discharge events at three different time scales revealed large-amplitude, high-frequency, and rhythmic activities (Fig. 5).

### Extended recording revealed distinct states of brain network activity

**Figure 6:**
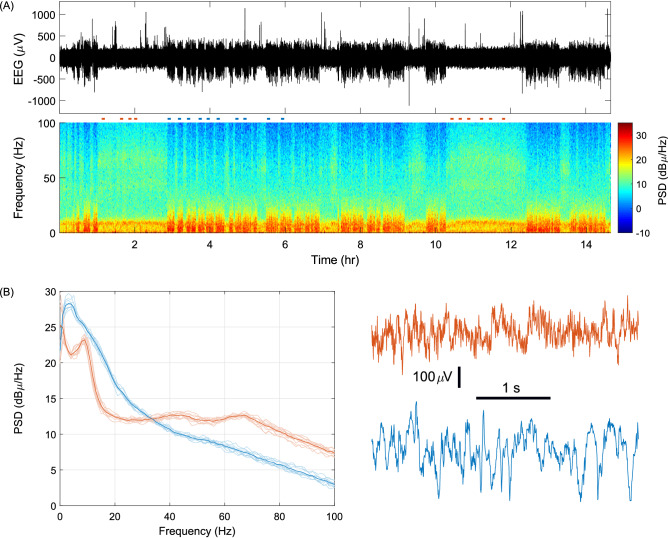
Extended recording revealed distinct states of brain network activity. (**A**) EEG signal recorded for more than 14 h starting at 8 pm and time–frequency analysis showing power spectral density (PSD) from 0 to 100 Hz over time. (**B**) Left: Power spectral density from 0 to 100 Hz of ten data segments, from both states, marked by corresponding colored dashes in (**A**). Thicker lines are the averages of the respective groups. Right: representative traces of raw EEG recording of the two groups.

We also tested OSERR for extended recording of more than fourteen hours. The system proved to be robust for extended recording at this time scale, demonstrated by continuous recording of EEG activity of good quality (Fig. 6A). The results revealed that the EEG activity seemed to alternate between two grossly distinct states with different power levels. Further examination with time–frequency analysis demonstrated that one state displayed increased power mostly in a low frequency range (Fig. 6A), potentially corresponding to sleeping periods of the animal, which are characterized by cortical EEG of higher amplitudes than awake stages^42,43^. In addition, power spectral analysis revealed that during the possibly sleeping state, there was a peak in the delta band (1–4 Hz, Fig. 6B), presumably corresponding to the stage of non-rapid eye movement sleep, the predominant form of sleep in mice^42,43^. The other state, conversely, had higher power in the gamma frequency band (30–100 Hz, Fig. 6B), presumably corresponding to the awake state^44^.

### Changes in EEG activity accompanying anesthesia induced by isoflurane inhalation

**Figure 7:**
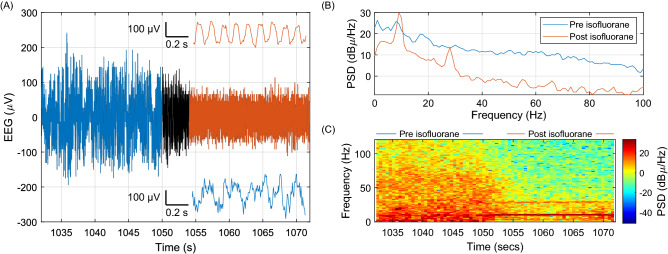
Changes in EEG activity accompanying anesthesia induced by isoflurane inhalation. (**A**) Representative EEG signal from one mouse. Insets show detailed views of segments from before (blue) and after (orange) anesthesia induction. (**B**) Power spectral density of the EEG signal before and after anesthesia induction. (**C**) Spectrogram showing the transition from pre- to post-anesthesia.

Next, we recorded changes in EEG activity accompanying anesthesia. After the subject mouse was connected to the standalone system, he was place in an induction chamber with 5% isoflurane in 100% O_2_. Anesthesia was rapidly induced and we observed robust changes in raw EEG signal (Fig. 7A), power spectral density (Fig. 7B), and spectrogram (Fig. 7C). Consistent with previous reports^45^, isoflurane exerted an inhibitory effect on the cortical network activity.

### Sex-dependent EEG activities during social interaction

**Figure 8:**
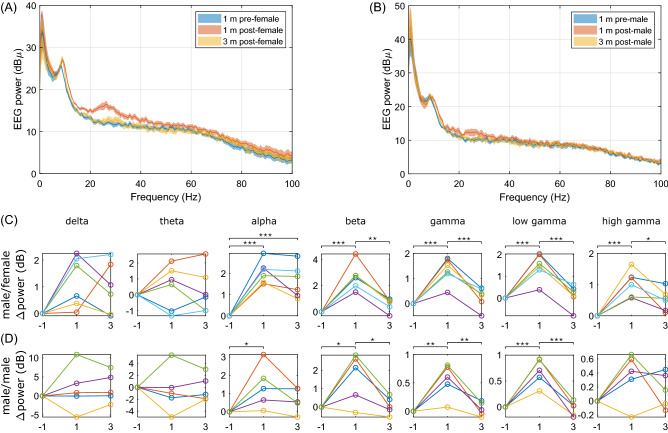
Sex-dependent EEG activities during social interaction. (**A**,**B**) Plots of mean ± SEM of power spectral density in last minute before (blue), first minute after (orange) and third minute after (yellow) the introduction of a female (**A**, n = 6) or male (**B**, n = 5) intruder to the home cage of the male subject mouse. (**C**,**D**) Plots of EEG power in delta to gamma bands, normalized to the power during the last minute before the introduction of the female (**C**) or male (**D**) intruder. To compare the EEG power in each band during the three time periods, one way repeated measures ANOVA was used, followed by multiple pairwise comparison if P < 0.05. For male–female interaction, delta band: P = 0.044; theta band: P = 0.829; alpha band: P < 0.001; beta band: P < 0.001; gamma band: P < 0.001; low gamma band: P < 0.001; high gamma band: P < 0.001. For male-male interaction, delta band: P = 0.383; theta band: P = 0.829; alpha band: P = 0.046; beta band: P = 0.014; gamma band: P = 0.002; low gamma band: P < 0.001; high gamma band: P = 0.119. *P < 0.05, **P < 0.01, ***P < 0.001 for post-hoc pairwise comparison.

As mentioned earlier, an advantage of OSERR is the potential of investigating brain activities of multiple interacting animals simultaneously, such as during social interaction. To explore this potential, we recorded EEG activity of a male resident mouse when a novel intruder mouse was introduced to the home cage of the subject. We found that changes in the EEG activity induced by the introduction of the intruder is sex-dependent. When comparing the power spectra of EEG activity during the last minute before versus the first minute after the introduction of a female intruder, there was an obvious increase in the EEG power. During the third minute after the introduction, the power spectrum largely returned to the before-introduction level (Fig. 8A). Conversely, the changes were subtler in case of a male intruder (Fig. 8B). We further calculated the EEG power in different bands, and results revealed that after either a female or male intruder was introduced, the EEG power in the alpha, beta, and gamma bands of individual subject was mostly increased during the first minute after the introduction, and reduced to pre-introduction levels during the third minute after the introduction except alpha band (Fig. 8C,D). However, the amplitude of increase was generally larger in the case of a female intruder, and occurred in both low and high gamma bands rather than only in low gamma band as in the case of a male intruder.

**Figure 9:**
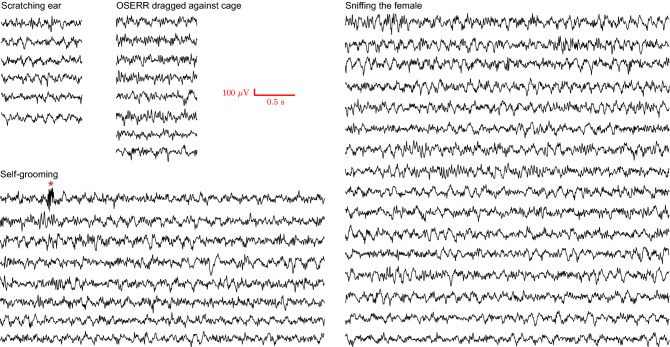
Examination of artifacts in EEG signal during different behaviors. Representative EEG traces recorded during the following activities of four subjects in male–female social interaction test: scratching ear, self-grooming, dragging the device against the cage and sniffing a female, which involved vigorous motion that was prone to cause motion artifacts. Only one instance of artifact was seen, marked by the red asterisk.

In this test, the subject animal tried to chase and sniff the intruder, resulting in close contact with the intruder and hitting the wall of the cage with OSERR multiple times. This led to the concern of artifacts in the recorded EEG signal. Therefore, we examined the EEG signal more closely during the following behavioral episodes for the presence of artifacts: (a) the subject mouse was scratching his ear (a behavioral reported to have high possibility to induce artifact in recording); (b) the subject was grooming himself; (c) the subject was dragging OSERR against the wall of the cage; (d) the subject was sniffing the intruder in close contact (Fig. 9). Representative videos that play EEG waveforms and simultaneously videotaped behaviors during each of these four behavioral episodes are shown in Supplementary Videos 1–4. Similarly, a video that plays EEG waveforms and convulsive movements during seizure is shown in Supplementary Video 5.

## Discussion

### Electrophysiological recording during behavior test to study brain function

Rodent models are essential for our understanding of brain function in health and disease. In vivo physiological recordings in rodent models are useful tools for dissecting pathways and mechanisms of how the brain works. In addition, in vivo recordings are also instrumental for drug development, such as testing the efficacy, pharmacodynamics/pharmacokinetics, and safety/toxicity in pre-clinical studies. However, restraining methods such as anesthesia or tethering can induce stress and thus affect basic physiological functions including hormonal levels, heart rate, blood pressure, body temperature, and food intake^27,28^. In general, untethered recordings from conscious animals are thought to be superior to those from restrained or anesthetized animals, since they represent a more normal physiological and behavioral state and are more predictive of the results that would be achieved in humans^27,28^. Furthermore, rapid advance in in vivo optogenetic manipulation and imaging of the brain has provided researchers unprecedented power, yet in most cases they require a fiber for transmitting light. To combine in vivo optogenetic manipulation or imaging with electrophysiological recording, a tethered system would be inconvenient. Radio telemetry overcomes the potential confounding issues with anesthesia and tethering, but requires a receiver relatively close to the subject to operate and may not be compatible with the arena needed.

Directing behavior is one of the most important functions of the nervous system^11–16^. Behavioral tests in rodent models are widely used and an integral part of neuroscience research. Although a large variety of species has been utilized for this purpose, mice and rats are most often used. They are easy to house and breed, with many outbred stocks and inbred strains readily available. More recently, increasingly sophisticated genetic manipulations in mice and rats have opened doors to understanding gene function, cellular processes, and circuit properties of the nervous system in health and disease, and to finding treatments. Measurement of behavioral outcomes is essential for this process^11–16^.

Over time, there has been a continuous effort to develop behavioral tests and there are well over 100 tests in contemporary use to evaluate traits such as sensory abilities, motor functions, learning and memory, and social interactions, as well as to model behaviors in psychiatric conditions including anxiety, depression, and addiction^11–16^. Although the protocols can be straightforward, behavioral tests are very sensitive to environmental factors. Recent improvements in technology including 3D printing have facilitated the design and rapid construction of sophisticated behavioral arenas and structures of a large variety of shapes and sizes. In addition, it has been proposed that behavioral testing in a more natural environment would provide ecologically relevant assessment, and have greater value in predicting human conditions the behavioral assays model. For example, a study described a new ethological enriched setting called PhenoWorld, which added significant discriminative power to screen depressive-like behavior^46^. In a separate study, an open source system called Eco-HAB for analysis of social activity was presented, which could measure spontaneous social behaviors in group-housed animals^47^. Both these studies utilized spacious and intricate testing arenas that are consisted of many components and subdivisions of various shapes and internal configurations, which were closer to the natural habitat of rodents.

Recording brain activity during animal behavior is a powerful technique that could offer a window to explore how brain function controls behavior in real time and live animals. The standalone system we present here, OSERR, would be very useful for this purpose. It was able to amplify, filter, digitize and store two channels of EEG signal at a 10-bit resolution and 240 Sps sampling rate for over 15 h. It weighs 1.8 g including the battery and occupies 13 × 8 × 8 mm^3^, suitable for even juvenile mice. Importantly, it eliminates the physical limitations of tethering or the requirement of a receiver. Thus, it is compatible with most behavioral settings and arenas, even the ones with complex structures mentioned above, as well as home cage monitoring^48^. It can also be waterproofed for assays conducted in water, such as the water maze test. In addition, it would be especially useful for simultaneously recording brain activities in a group of animals, which would be instrumental for studies involving social interaction in health and disease, such as autism, for which atypical social interaction is a hallmark and is hypothesized to involve altered connectivity and imbalanced network activity^49–58^.

Another advantage of OSERR is its resistance to motion artifact in the recorded data. For example, our recording had minimal motion artifact during (a) the test of induced seizure, in which the animal fell multiple times and displayed robust convulsions (Fig. 5 and Supplementary Video 5), and (b) social interaction tests, in which the subject animal tried to chase and sniff the intruder, and also did ear-scratching and self-grooming, and dragged OSERR against the wall of the cage (Fig. 9 and Supplementary Videos 1–4). Thus, the data would be more compatible with analysis using algorithms instead of manual detection and quantification, resulting in more efficient and objective measurement.

A noteworthy feature of OSERR is that it is open source. The design of the hardware and software for the purpose of data collection and conversion is freely available. In addition, this system is adaptable and flexible, can be adjusted to encompass new recording paradigms such as local field potential by depth electrodes and even unit recordings.

### Limitations of OSERR and future directions

There are two main limitations in the electronic design of the system: the maximum recording duration and the limited number of channels. As outlined in the results section, currently the recording duration is limited by the size of the onboard memory to 15.5 h. Based on the power consumption, the system can run for over 72 h with the existing battery. Thus, the recording duration could be extended by using a larger memory. Memories over 2 Gbit are available in the same physical size as the one used, enabling operation for over 72 h. Further increase would be possible at the cost of a physically larger and heavier system design. The system has two differential channels, which could be used for recording surface EEG, local field potential with depth electrodes, or electromyography. Further channels could be added within the same physical size by simplifying the analog design, e.g. implementing lower order filters outside the passband which will require fewer components. If the physical size constraint can be relaxed, another option is to simply duplicate the current design. Both will require significant changes in the schematic and PCB designs. Our belief is that with the open-source documentation, the community can adapt the design to their specific needs. We believe this is also what separates this work from commercially available solutions that are not easily customizable. The bandwidth and gain of OSERR can be set by choosing appropriate resistor and capacitor values. This is ultimately limited by the gain bandwidth product of the opamps used. In simulation, we were able to go up to a bandwidth of 300 Hz with a gain of 1000, suitable for field potential recording. The sampling rate can be set in firmware, limited by the microcontroller’s analog to digital convertor, to well over 10 kSps.

OSERR has an on-board LED that flashes at the start of data acquisition. This flash can be captured on a single frame on a camera, thus synchronizing the EEG acquisition with the video recording (Fig. 2B). Temporal resolution of the synchronization is limited by the framerate of the camera. Typical 30 fps videos would limit synchronization resolution to 33 ms, sufficient to synchronize with most other recording or stimulation paradigms, such as behavioral recording, photometry signal, and chemogenetic or optogenetic stimulation. However, it will not be adequate for synchronizing OSERR with events on millisecond scale, such as spike recording. A potential concern with this method is that it is susceptible to error and drift in the sampling rates of the individual devices. However, this can be calibrated for and the OSERR firmware can be modified to emit flashes at regular intervals rather than only at startup. In addition, due to the design to achieve complete self-containment, certain compromises have been made and the synchronization procedure is more cumbersome than communicating directly with an external trigger, for example.

Other trade-offs include the absence of real-time data streaming; data are recorded and stored in the device, and can only be transferred after the recording has completed. Real-time quality checking of recorded signal can be very important, especially for certain experiments such as ones involving a large amount of time and effort for each recording. With the device presented here, there are two ways to examine the signal quality before an experiment: (1) plug the device onto the animal, connect the analog output of the device (for example, by using fine clips) to an oscilloscope and view the signal in real time; (2) plug the device onto the animal, record for a brief period of time, and then unplug the device and view the recorded data using the MATLAB interface included in the open-source documentation, which will only take a few minutes to transfer, save, and visualize the signal. Notably, as the device connects to the electrodes through a connector, and both the electrodes and connector are cemented in place, plugging/unplugging the device will not harm signal quality.

Another limitation of the current version is that it does not implement turning off and restarting recording. In addition, presently the LED is flashed once at the start of recording. With small modifications to the software, turning off and restarting recording could be achieved, and the LED could be flashed every N samples to act as a status monitor. Furthermore, like any other implant-based method, use of OSERR could potentially affect animal behavior, due to factors such as inflammation caused by surgeries, and impediments to physical movement or even injury when the headstage is caught in meshes or wires used in the housing cage or testing arena.

The development of in vivo electrophysiological recording systems has advanced rapidly in recent years, reflecting the high demand for those devices and their usage in diverse areas of neuroscience research. Depending on the requirements of the experiment, the advantages and disadvantages of different recording systems need to be carefully considered. As mentioned earlier, tethered systems can record from a large number of channels with high sampling frequencies, suitable for recording neuronal ensembles or high-resolution activity mapping in both global and local brain areas^23–25^. Telemetry systems offer the versatility of being wireless and restraint-free, and are less prone to induce injuries in the subjects^26–30^. Both tethered and telemetry systems have the ability to stream data in real-time for visualization and closed-loop feedback. Standalone systems are advantageous in the wild or test arenas whose shape or size hinder signal transmission between the recording device and the receiver, but have to balance channel count, sampling frequency, memory storage, and battery life due to constraints on the weight and size of the device.

## Conclusion

In this study, we developed OSERR, an open-source standalone electrophysiology recording system for rodents, and demonstrated its utility in several biological conditions. It is compatible with a variety of arenas and settings in diverse behavioral assays, and the open-source documentation enables the researchers to customize the design of the system to their own needs. Complementary to the existing technologies, OSERR could help to further the investigation of behavior and brain activity.

## Supplementary information

Supplementary Information 1.

Supplementary Video S1.

Supplementary Video S2.

Supplementary Video S3.

Supplementary Video S4.

Supplementary Video S5.

Supplementary Information 2.
