# Supplementary material for: OSERR: an open-source standalone electrophysiology recording system for rodents
Source: Sci Rep. 2020 Oct 12;10:16996. doi: 10.1038/s41598-020-73797-4 (PMC7552399; doi:10.1038/s41598-020-73797-4)
Supplement: Supplementary file 1 — Supplementary Information 1. [file 41598_2020_73797_MOESM1_ESM.pdf]

OSERR: an open-source standalone electrophysiology recording system for rodents

Ning Cheng[1] and Kartikeya Murari[2]

[1]Alberta Children's Hospital Research Institute (ACHRI), Cumming School of Medicine, University of Calgary, Calgary, Alberta, Canada

[2]Department of Electrical and Computer Engineering, Schulich School of Engineering, University of Calgary, Calgary, Alberta, Canada

This document contains a listing of the supplementary files along with brief descriptions.

`OSERR_operation.pdf` This file describes the overall operation of OSERR while recording data and also while reading the data using the USB/OSERR interface board. It also includes the logic used in the firmware running on the microcontroller and the software used to read data from OSERR.

`OSERR_schematic.pdf` This file has the circuit schematics of OSERR. The component values indicated are what were used for the system described in the paper.

`OSERR_partsList.pdf` This file has a list of all components used in OSERR and in the USB/OSERR interface board. Manufacturer part numbers and current prices are included for everything except for the passive components.

`OSERR_layout_assembly.pdf` This file has the PCB layout for OSERR and an assembly guide.

`USB_OSERR_InterfaceBoard_schematic.pdf` This file has the circuit schematics of USB/OSERR interface board.

`sourcecode\microcontrollerCode.asm` This file has the assembly source code for the firmware running on the microcontroller.

`sourcecode\usb2SPI-matlabUI.c` This file has the C source code for interacting with the USB/OSERR interface board. It is not used directly.

`sourcecode\OSERR.m, sourcecode\OSERR.fig` These two files constitute the MATLAB graphics user interface that use the C application above for interacting with the USB/OSERR interface board.

`PCBfabFiles\` This directory contains the Gerber files and the NC drill file needed for PCB fabrication

Supplementary video 1: EEG waveform and simultaneously videotaped ear-scratching behavior of the subject animal

Supplementary video 2: EEG waveform and simultaneously videotaped self-grooming behavior of the subject animal

Supplementary video 3: EEG waveform and simultaneously recorded video of the subject animal dragging OSERR against the wall of the cage

Supplementary video 4: EEG waveform and simultaneously recorded video of the subject animal chasing and sniffing the intruder in close contact

Supplementary video 5: EEG waveform and simultaneously videotaped convulsive movements of the subject animal during seizure
.
