# Supplementary figures and images for: OSERR: an open-source standalone electrophysiology recording system for rodents
Source: Sci Rep. 2020 Oct 12;10:16996. doi: 10.1038/s41598-020-73797-4 (PMC7552399; doi:10.1038/s41598-020-73797-4)

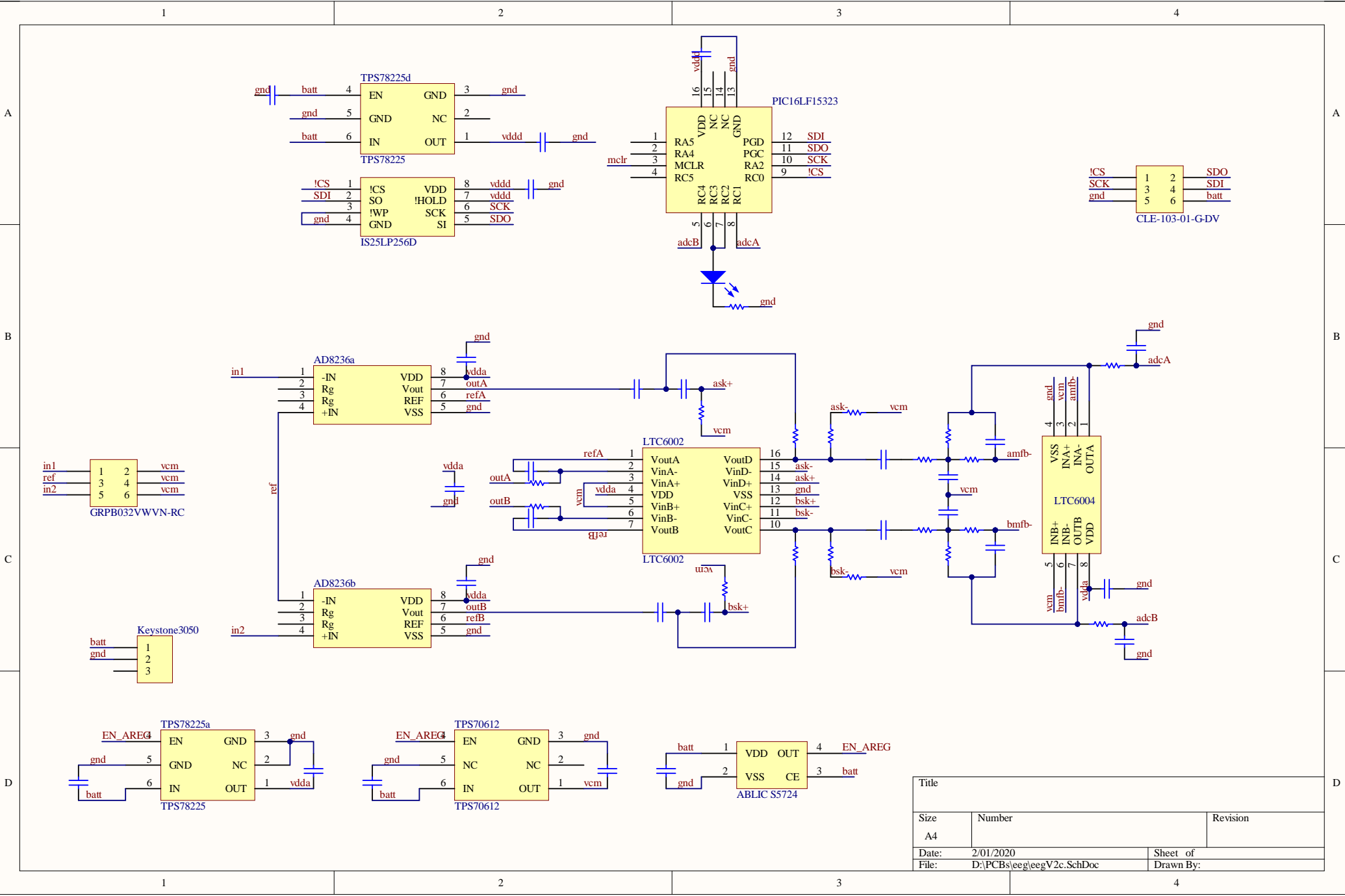

|                                   |           |          |
|-----------------------------------|-----------|----------|
| Title                             |           |          |
| Size<br>A4                        | Number    | Revision |
| Date:<br>2/01/2020                | Sheet of  |          |
| File:<br>D:\PCBs\eegeegV2c.SchDoc | Drawn By: |          |

Supplement: Supplementary file 7 — Supplementary Information 2. [file 41598_2020_73797_MOESM7_ESM.tar]
